# Detection of *BRAF* Mutations Using a Fully Automated Platform and Comparison with High Resolution Melting, Real-Time Allele Specific Amplification, Immunohistochemistry and Next Generation Sequencing Assays, for Patients with Metastatic Melanoma

**DOI:** 10.1371/journal.pone.0153576

**Published:** 2016-04-25

**Authors:** Alexandre Harlé, Julia Salleron, Claire Franczak, Cindy Dubois, Pierre Filhine-Tressarieu, Agnès Leroux, Jean-Louis Merlin

**Affiliations:** 1 Université de Lorraine, Faculté de Pharmacie, Nancy, France; 2 CNRS UMR 7039 CRAN, Nancy, France; 3 Institut de Cancérologie de Lorraine, Service de Biopathologie, Vandoeuvre-lès-Nancy, France; 4 Institut de Cancérologie de Lorraine, Cellule biostatistique, Vandoeuvre-lès-Nancy, France; University of Navarra, SPAIN

## Abstract

**Background:**

Metastatic melanoma is a severe disease with one of the highest mortality rate in skin diseases. Overall survival has significantly improved with immunotherapy and targeted therapies. Kinase inhibitors targeting BRAF V600 showed promising results. *BRAF* genotyping is mandatory for the prescription of anti-BRAF therapies.

**Methods:**

Fifty-nine formalin-fixed paraffin-embedded melanoma samples were assessed using High-Resolution-Melting (HRM) PCR, Real-time allele-specific amplification (RT-ASA) PCR, Next generation sequencing (NGS), immunohistochemistry (IHC) and the fully-automated molecular diagnostics platform Idylla^TM^. Sensitivity, specificity, positive predictive value and negative predictive value were calculated using NGS as the reference standard to compare the different assays.

**Results:**

*BRAF* mutations were found in 28(47.5%), 29(49.2%), 31(52.5%), 29(49.2%) and 27(45.8%) samples with HRM, RT-ASA, NGS, Idylla^TM^ and IHC respectively. Twenty-six (81.2%) samples were found bearing a c.1799T>A (p.Val600Glu) mutation, three (9.4%) with a c.1798_1799delinsAA (p.Val600Lys) mutation and one with c.1789_1790delinsTC (p.Leu597Ser) mutation. Two samples were found bearing complex mutations.

**Conclusions:**

HRM appears the less sensitive assay for the detection of *BRAF* V600 mutations. The RT-ASA, Idylla^TM^ and IHC assays are suitable for routine molecular diagnostics aiming at the prescription of anti-BRAF therapies. Idylla^TM^ assay is fully-automated and requires less than 2 minutes for samples preparation and is the fastest of the tested assays.

## Introduction

Metastatic melanoma is a severe disease with one of the highest mortality rate in skin diseases [1]. Response rate, progression-free survival and overall survival have significantly improved these last years with the development of immunotherapy and targeted therapies. Kinase inhibitors specifically targeting BRAF with V600 mutated phenotype, like vemurafenib or dabrafenib showed promising results compared to dacarbazine chemotherapy [2, 3]. The association of dabrafenib with an anti-MEK therapy *i*.*e*. trametinib, also showed a significantly higher overall survival than vemurafenib alone in *BRAF* mutated patients with metastatic melanoma [4]. Since these anti-BRAF targeted therapies are only effective on *BRAF* V600 mutated melanomas, the detection of *BRAF* mutations on exon 15, especially on the V600 hotspot is mandatory for prescription. BRAF mutations are found in approximately 50% of metastatic melanomas [5] with V600E (c.1799T>A; p.Val600Glu), V600K (c.1798_1799delinsAA; p.Val600Lys), V600R (c.1798_1799delinsAG; p.Val600Arg) and V600M (c.1798G>A; p.Val600Met) in 79%, 12%, 5% and 4% frequencies respectively.

Most of the assays for the routine detection of *BRAF* mutations in metastatic melanomas are PCR-based, but sequencing or immunohistochemistry assays are also widely used [6, 7]. The ideal assay should be easy, accurate and highly sensitive to ensure the detection of low *BRAF* mutant allele frequency.

In this study, we compared the new Idylla^TM^ fully-automated CE-IVD platform with four routine assays in our lab. Fifty-nine samples from patients with metastatic melanoma were assessed for *BRAF* mutations using Idylla^TM^, high resolution amplicon melting (HRM), real-time allele specific amplification (RT-ASA), next generation sequencing (NGS) and immunohistochemistry (IHC).

## Materials and Methods

### Patients and samples

Fifty-nine formalin-fixed paraffin-embedded tumor samples from patients treated for a metastatic melanoma at Institut de Cancérologie de Lorraine between 2012 to 2015 have been retrospectively collected for this study. All patients gave their consent for the research of *BRAF* mutations and the use of their samples. Study has been approved by Institut de Cancérologie de Lorraine scientific board. All patients’ data have been anonymized and de-identified prior to analysis. Tumor specimens were macrodissected after hematoxylin-eosin slide examination by a qualified senior pathologist to evaluate the percent tumor nuclei in the sample selected for DNA extraction [8]. For HRM, RT-ASA and NGS assays, one macrodissected section of ten micrometer was used for DNA extraction with no restriction of tumor cell content. For Idylla^TM^ assay, one ten micrometer section from FFPE samples was used. According to Idylla manufacturer’s recommendations, the sample introduced in the cartridge must contain at least 50% of tumoral cells with an area included in the 25-300mm² range. Multiple sections of 10μm and macrodissection has been used for samples that do not meet these criteria to ensure a total content of 50% of tumor cells. Samples characteristics are detailed in **Table 1**.

**Table 1:** Samples characteristics.

Sample #	Localization	Percent tumor nuclei	DNA concentration (ng/μL)
**1**	right side	90%	149.3
**2**	axillary node	90%	54.5
**3**	axillary node	90%	23.3
**4**	elbow nodule	90%	29.5
**5**	left foot	95%	27.3
**6**	axillary node	90%	17.0
**7**	left thigh	90%	52.0
**8**	left arm	90%	99.5
**9**	inguinal lymph node	80%	17.5
**10**	sentinel node	20%	118.2
**11**	node	90%	73.4
**12**	cervix	90%	20.8
**13**	node	90%	61.3
**14**	right buttock	90%	22.5
**15**	axillary node	60%	22.0
**16**	mastoid	80%	51.5
**17**	breast	90%	107.3
**18**	heel	40%	33.5
**19**	scapular bone	80%	130.8
**20**	heel	30%	10.4
**21**	right arm	20%	33.4
**22**	node	90%	16.3
**23**	iliac node	90%	55.8
**24**	cheek	70%	10.6
**25**	inguinal lymph node	90%	31.0
**26**	arm node	95%	20.5
**27**	axillary node	30%	115.8
**28**	lumbar vertebrae	10%	2.1
**29**	pubic symphysis	90%	145.5
**30**	inguinal lymph node	60%	155.0
**31**	inguinal lymph node	90%	52.0
**32**	scapular bone	75%	103.5
**33**	inguinal lymph node	90%	89.0
**34**	inguinal lymph node	30%	114.5
**35**	foot nodule	90%	6.8
**36**	gluteal crease	80%	30.5
**37**	pectoral muscle	90%	12.9
**38**	right calf	90%	169.8
**39**	inguinal lymph node	20%	64.8
**40**	left thigh	10%	5.4
**41**	axillary node	95%	109.3
**42**	maxillofacial	80%	54.2
**43**	right thigh	90%	40.5
**44**	right arm	75%	65.8
**45**	axillary node	40%	121.7
**46**	axillary node	50%	56.3
**47**	scapular bone	90%	51.0
**48**	abdominal lesion	85%	16.5
**49**	sentinel node	60%	39.8
**50**	scalp	60%	20.2
**51**	axillary node	20%	62.5
**52**	vagina	50%	76.5
**53**	axillary node	50%	127.5
**54**	back	25%	36.2
**55**	iliac node	90%	82.3
**56**	right arm	70%	66.5
**57**	axillary node	90%	23.8
**58**	anal margin	70%	17.8
**59**	right arm	50%	30.3

### DNA isolation

For HRM, RT-ASA and NGS, DNA isolation was performed using COBAS® DNA Sample preparation kit (Roche Diagnostics, Meylan, France), as described in the manufacturers protocol. DNA was finally eluted in a volume of 100μL of buffer. DNA concentrations were assessed in duplicate using NanoVue spectrophotometer (GE Healthcare, Buc, France). For Idylla^TM^, DNA was automatically extracted in the cartridge. No DNA extraction was needed for IHC.

### *BRAF* mutation detection

**Fig 1:**
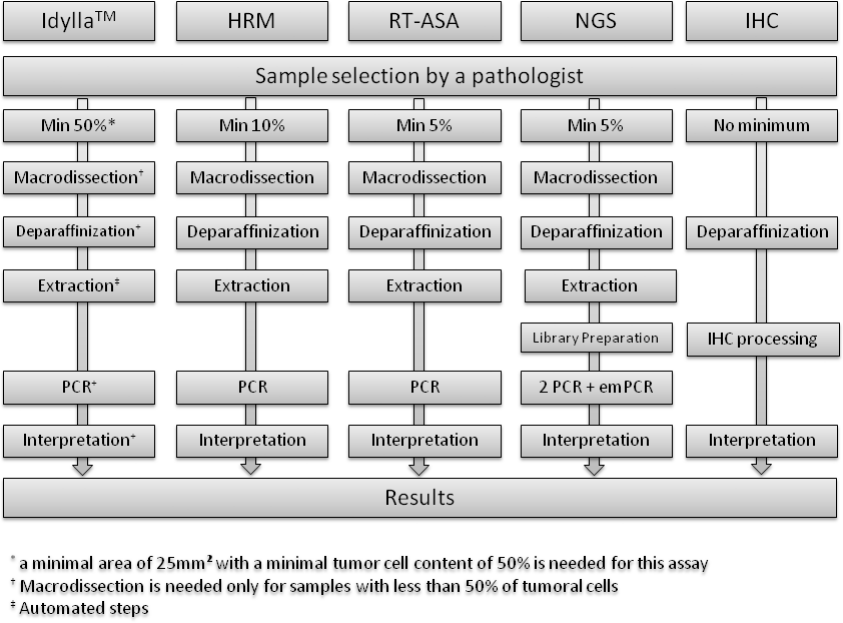
Workflows for the different assays used for *BRAF* mutation analysis.

All workflows are described in **Fig 1.**

#### Idylla^TM^ platform

Idylla^TM^ platform (Biocartis, Mechelen, Belgium) is a fully catridge-based automated platform and uses microfluidics processing with all reagents on-board. The platform comprises a console and up to eight independent processing units allowing the detection of 8 samples at the same time (*e*.*g*. *BRAF*, *KRAS*, *NRAS* or *EGFR* mutations in different samples). All 59 melanoma samples were assessed for the detection of *BRAF* p.V600E (c.1799T>A; p.Val600Glu), p.V600E2 (c.1799_1800delinsAA; p.Val600Glu), p.V600D (c.1799_1800delinsAT and c.1799_1800delinsAC; p.Val600Asp), p.V600K (c.1798_1799delinsAA; p.Val600Lys), p.V600R (c.1798_1799delinsAG; p.Val600Arg) and p.V600M (c.1798T>A; p.Val600Met) mutations. Macrodissected FFPE samples sections were transferred to wetted (nuclease-free water) filter papers. A second wetted filter paper was then added on top of the FFPE material. Sample with the two wetted filter papers was finally placed on the lysis pad in the Idylla^TM^ BRAF mutation test cartridge (Biocartis) and inserted in the instrument. Inside the cartridge, the sample is homogenized and cells lysed using a combination of high intensity focused ultrasound, enzymatic/chemical digestion and heat. The nucleic acids are liberated and ready for subsequent PCR amplification. The PCR is real-time and uses a fluorophore-based detection system.

After a 90 minutes run, all steps were automatically performed inside the cartridge and final reports were directly available on the system after an automatic on-board post-PCR curve analysis. *BRAF* V600E, V600E2 and V600D were detected as “V600E/E2/D Mutation” and V600K, V600R and V600M as “V600K/R/M Mutation” by the system. The system had been previously calibrated using BRAF V600E, V600K and wild-type standards (Horizon Diagnostics).

#### High Resolution Melting

HRM analysis was performed using the LC 480 HRM Master kit (Roche Diagnostics, Meylan, France) and 96 well plates (Roche Diagnostics) using LC480 thermocycler (Roche Diagnostics) as previously described [9, 10]. Melting curves were analyzed using LightCycler 480 software v.1.5. (Roche Diagnostics).

DNA extracted from WidR cell line (ATCC® CCL-218^TM^) was used as *BRAF* V600E (c.1799T>A; p.Val600Glu) mutated positive control and DNA extracted from LoVo cell line (ATCC® CCL-229^TM^) was used as *BRAF* wild-type control.

#### Real-time allele specific amplification

Real-time allele specific amplification analysis was performed using the LC 480 SybrGreen Master kit (Roche Diagnostics) and 384 well plates (Roche Diagnostics) using LC480 thermocycler (Roche Diagnostics) as previously described [9, 11]. Specific primers for the detection of BRAF p.V600E (c.1799T>A; p.Val600Glu), p.V600K (c.1798_1799delinsAA; p.Val600Lys), p.V600R (c.1798_1799delinsAG; p.Val600Arg) and p.V600D (c. 1799_1800delinsAT; p.Val600Asp) were used for this study.

DNA extracted from WidR cell line was used as *BRAF* V600E (c.1799T>A; p.Val600Glu) mutated positive control and DNA extracted from LoVo cell line was used as *BRAF* wild-type control. DNA extracted from standard FFPE samples (Horizon Diagnostics, Cambridge, UK) were used for V600K (Horizon HD268) and V600R (Horizon HD275) mutated positive controls. No positive control was used for V600D mutation because of the rarity of this mutation.

#### Next Generation Sequencing

Ultra-deep pyrosequencing (Roche Diagnostics) was used to detect mutations on exon 15 of BRAF. Fifty nanograms of DNA were used for PCR amplification (High Fidelity PCR System, Roche Diagnostics, Meylan, France) with specific primers (Forward 5’- ACCTAAACTCTTCATAATGCTTGCT-3’; Reverse 5’-AACTCAGCAGCATCTCAGGG-3’) designed using Primer3Plus online software v.2.3.6 [12]. Universal M13 tails (Forward and Reverse) were added to the specific designed primers for the amplification of the target regions (exon 15 of BRAF). A second PCR was then assessed including multiplex identifiers (MIDs), adaptators and complementary sequence of the universal tail used for the first PCR. Quality of synthesized amplicons was assessed using 1% agarose gel for proper amplification. Amplicon processing was done as described by the Amplicon Library Preparation and emulsion PCR (emPCR; Lib-A) Method GS Junior Titanium Series manual from Roche Diagnostics. Amplicons were purified using Agencourt AMPure XP beads (Beckman Coulter, SA, Nyon, Switzerland) and High Pure PCR Product purification kit (Roche Diagnostics) and finally quantified with the Quant-it TM PicoGreen dsDNA Assay Kit (Life Technologies, Oregon, USA). An emulsion PCR (emPCR) was finally assessed with 1.10^6^ molecules, and 5.10^6^ enriched beads were loaded on a picotiter plate for GS Junior Sequencer (Roche Diagnostics). Data were treated with Amplicon Variant Analyzer software (454 Life Sciences Corp. Roche, Branford, Connecticut, USA). Sequences were aligned with NM_004333 for reference sequence and variant calling was processed. At X1000 depth, NGS sensitivity was 1%. A second data analysis using SeqPilot (SeqNext, JSI medical systems, Ettenheim, Germany) was performed. A final analysis using BWA 0.7.12 (mem algorithm, default parameters) for mapping and sorting and indexing using SAMtools was performed. VarScan2 (mpileup2snp algorithm, with filters—min-coverage 100—minreads 20—min-var-freq 0.01—p-value 0.05) was used for variant calling [13].

#### Immunohistochemistry

**Fig 2:**
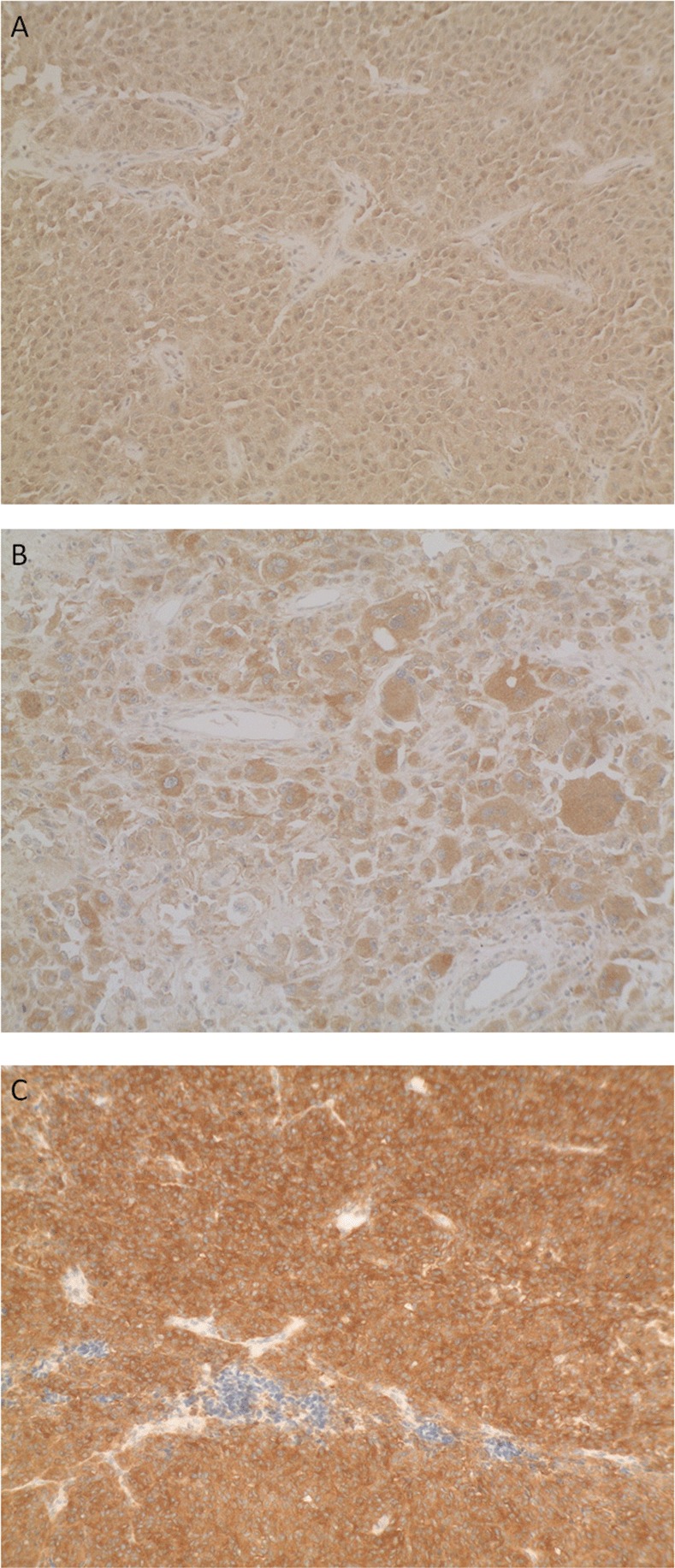
Immunohistochemistry staining according to *BRAF* mutational status. No signal can be observed in BRAF wild-type sample (**A**), but medium (**B**) to strong signal (**C**) is observed in samples presenting a BRAF V600E mutation.

IHC was assessed on 5μm tissue sections from the same tissue block used for mutation testing and VE1 V600E specific antigen (Spring Bioscience, Pleasanton, CA, USA) diluted at 1/50 was used as previously described [14]. All process was automated using BenchMark Ultra (Ventana, Meylan, France). Briefly, staining procedures included sample deparaffinization using EasyPrep (Roche Diganostics), drying at 72°C for 15 minutes, pretreatment with cell conditioning 1 pH 8 (Roche Diagnostics) for 52 minutes and incubation with the primary antibody at 42°C for 44 minutes. Antibody incubation was followed by counterstaining with one drop of hematoxylin for 12 minutes and one drop of bluing reagent for 4 minutes. For chromogenic detection, OptiView DAB IHC Detection Kit (Roche Molecular Diagnostics) was used as previously described [15, 16]. Slides were finally washed using detergent and mounted for observation. Two BRAF V600E-positive and one BRAF V600E-negative malignant melanoma confirmed samples were used as controls. Each run also contained buffer with no primary antibody as negative control. Staining scoring was finally assessed by a pathologist considering granulocytoplasmic intensity (**Fig 2**). Staining was defined as 0+ staining intensity when comparable to negative BRAF V600E-negative control sample. Low, moderate and strong cytoplasmic staining intensities were defined as 1+, 2+ and 3+, respectively. All scoring were assessed blinded to other outcomes.

### Statistical analysis

Qualitative parameters were described by frequency and percentage. Specificity (Sp), sensitivity (Se), positive predictive value (PPV) and negative predictive value (NPV) were calculated using NGS as the reference standard.

Statistical analysis was performed using SAS software (SAS Institute, Cary, NC 25513; version 9.2).

## Results

All samples were processed using the 5 assays and provided interpretable results in all cases. Mutations on BRAF were found in 28 (47.5%), 29 (49.2%), 31 (52.5%), 29 (49.2%) and 27 (45.8%) samples with HRM, RT-ASA, NGS, Idylla^TM^ and IHC respectively (**Table 2**). Twenty-six (81.2%) samples were found bearing a c.1799T>A (p.Val600Glu) mutation, three (9.4%) with a c. 1798_1799delinsAA (p.Val600Lys) mutation and one with c.1789_1790delinsTC (p.Leu597Ser) mutation. Two samples were found bearing complex mutations. NGS data suggest that sample #40 contains 3 different tumoral clones: one clone bearing c.1796C>T (p.Thr599Ile) mutation, one clone bearing c.1799T>A (p.Val600Glu) mutation and one clone bearing both mutations. Sample #49 also bears a complex mutation c.1797delinsTACT (p.Tthr599delinsThrThr). Mutations details are presented in **Table 3**.

**Table 2:** Mutation results per assay.

	Wild-type	(%)	*BRAF* mutation*	(%)
High Resolution Melting	31	(52,5)	28	(47,5)
Real-time allele specific amplification	30	(50,8)	29	(49,2)
Next Generation Seqencing	28	(47,5)	31	(52,5)
Idylla^TM^	30	(50,8)	29	(49,2)
Immunohistochemistry	32	(54,2)	27	(45,8)

*BRAF mutations are detailed in Table 3

**Table 3:** Mutations details.

Mutations				Number of samples with mutation				
CDS mutation	AA variation	Cosmic ID	Frequency*	HRM^†^	RT-ASA	NGS	Idylla^TM^	IHC
c.1799T>A	p.Val600Glu	COSM476	80~90%	21	25	25	25	24
c.1798_1799delinsAA	p.Val600Lys	COSM473	8%	3	3	3	3	2
c.1789_1790delinsTC	p.Leu597Ser	COSM1126	< 1%	1	0	1	0	0
c.1796_1799delinsTAGA	p.Thr599_Val600delinsIleGlu	n/a	n/a	1	1	1	1	1
c.1797delinsTACT	p.Thr599delinsThrThr	n/a	n/a	1	0	1	0	0

*According to COSMIC database

^†^ One sample is considered as false positive with HRM and is not mentioned in this table Sp, Se, PPV and NPV for each of the four validated assays are summarized in Table 4. Lower specificity and PPV were seen when using HRM. Idylla^TM^ and RT-ASA yielded the highest sensitivity and NPV. Based on V600 cases, calculated Se, Sp, PPV and NPV were 100% for Idylla^TM^ and RT-ASA and 93.1%, 100%, 100% and 93.8% for IHC for Se, Sp, PPV and NPV respectively.

**Table 4:** Performance statistics of the BRAF detection with the four validated assays compared to NGS.

	HRM	RT-ASA	Idylla^TM^	IHC
Sensitivity	87.1%	93.5%	93.5%	81.7%
Specificity	96.4%	100.0%	100.0%	100.0%
PPV	96.4%	100.0%	100.0%	100.0%
NPV	87.1%	93.3%	93.3%	87.5%

Abbreviations: IHC, immunohistochemistry; NGS, next-generation sequencing; HRM: High Resolution Melting; RT-ASA: Real-Time Allele Specific Amplification; NPV, negative predictive value; PPV, positive predictive value.

Ten samples were found with discrepancies between the 5 assays (**Table 5**). Samples #1, #10 and #39 were identified as wild-type using HRM assay and as mutated with all the other assays. Sample #36 was found wild-type with HRM and IHC assays and bearing a mutation with the other assays. Sample #21 was found bearing a mutation with HRM assay and wild-type with all other assays. Samples #40 and #47 were found bearing a mutation with HRM and NGS assays and wild-type with the other assays. Sample #28 was found wild-type with IHC and mutated with the other assays. Finally, samples #16 and #50 have been found bearing a V600E with IHC and a V600K mutation with all the other assays.

**Table 5:** Samples with discrepancies.

Sample #	Tumoral cells content (%)	Mutant allele frequency (%)	Mutation*	Results			
				HRM	SybrGreen	Idylla	IHC
**1**	90	84.22	c.1799T>A p.Val600Glu	wild-type	V600E	V600E-E2-D	V600E
**10**	20	1.86	c.1799T>A p.Val600Glu	wild-type	V600E	V600E-E2-D	V600E
**16**	80	58.23	c.1798_1799delinsAA p.Val600Lys	Mutation on exon 15	V600K	V600K-R-M	V600E
**21**	20	-	wild-type	Mutation on exon 15	wild-type	wild-type	wild-type
**28**	10	7.59	c.1798_1799delinsAA p.Val600Lys	Mutation on exon 15	V600K	V600K-R-M	wild-type
**36**	80	39.44	c.1799T>A p.Val600Glu	wild-type	V600E	V600E-E2-D	wild-type
**39**	20	5.38	c.1799T>A p.Val600Glu	wild-type	V600E	V600E-E2-D	V600E
**40**	10	4.87	c.1796_1799delInsTAGA p.Thr599_Val600delinsIleGlu	Mutation on exon 15	wild-type	wild-type	wild-type
**47**	90	74.25	c.1789_1790delinsTC p.Leu597Ser	Mutation on exon 15	wild-type	wild-type	wild-type
**50**	60	37.11	c.1798_1799GT>AA p.Val600Lys	Mutation on exon 15	V600K	V600K-R-M	V600E

*Mutations characterized and confirmed by NGS

## Discussion

*BRAF* genotyping is important for the treatment of patients with metastatic melanoma. The use of anti-BRAF drugs (vemurafenib or dabrafenib) associated or not with anti-MEK therapy (trametinib or cobimetinib), has considerably improved progression-free and overall survival for patients bearing mutated V600 metastatic melanoma [4, 17]. In this study, we compared the new Idylla^TM^ BRAF Mutation test with standard methods already compared in some studies for the detection of *BRAF* somatic mutations [6, 18].

Numerous pre-analytical parameters may influence molecular mutations detection. The theoretical analytical sensitivities used in this study were 10% for HRM and 1% for Idylla^TM^, RT-ASA, IHC and NGS. Idylla^TM^ and RT-ASA yielded the highest sensitivity (both 93.5% and 100% when considering only V600 mutated samples). IHC yielded a 81.7% sensitivity when calculated with all the samples, but yielded 93.1% for V600 mutated samples which is conform with previously published data [19, 20]. Calculated specificity for Idylla^TM^, RT-ASA and IHC where 100%. Se and Sp for HRM were 87.1% and 96.4% respectively and was the less accurate assay for the detection of *BRAF* mutation on exon 15. The interpretation of HRM curves requires standardization and interpretation is sometimes difficult for samples with low quality DNA. Some false positive can be observed with IHC because of melanin pigmentation but a simple treatment with alkaline phosphatase can prevent this issue. No false positive sample was found with IHC in this study. No false positive sample was found in this study with PCR assays except for sample #21 which has been identified as mutated with HRM and as wild-type with all the other assays. We confirmed that no mutation was present in this sample by retesting it with all assays and NGS depth was set above 9500x. Samples #1, #10 and #39 were found as wild-type with HRM and are considered as false negative. Mutant allele/wild-type allele frequencies were 84.22%, 1.86% and 5.38% for these samples respectively and tumor cells proportion were 90%, 20% and 20% respectively. The lack of sensitivity of HRM assay can probably explain the false-negative results for samples #10 and #39, but cannot explain the results obtained for sample #1. We think that the lack of DNA quality of this sample did not allow the detection of the mutation.

Ten samples were found with discrepancies. Samples #40, #47 and #49 were found with c.1797delinsTACT (p.Thr599delinsThrThr), c.1789_1790delinsTC (p.Leu597Ser) and c.1796_1799delinsTAGA (p.Thr599_Val600delinsIleGlu) respectively. The c.1797delinsTACT (p.Thr599delinsThrThr) and c.1789_1790delinsTC (p.Leu597Sser) mutations were only detected with HRM and NGS which is obvious according to other assays technology based on the specific detection of V600 mutations. Samples #16, #28 and #50 were found bearing a c.1798_1799delinsAA p.Val600Lys mutations and surprisingly, samples #16 and #50 were found as V600E positive samples using IHC whereas the specificity of the VE1 BRAF V600E specific antigen should not allow the detection of these two cases as it did not for sample #28. Cross-reactivity has already been described with detection of BRAF V600E and V600R mutation [19]. We also hypothesize that the high percentage of tumoral cells in these two samples, 80% and 60% for samples #16 and #50 respectively, may allow the detection of low-V600E subclones in these samples. The low percent of tumor nuclei in sample #28 (10%) may have an impact on the no detection of potential of low-V600E subclones, or may simply prevent cross-reactivity. Finally, sample #36 was found wild-type with IHC and bearing a *BRAF* Val600Glu mutation with all other assays. Whereas the total proportion of tumor nuclei in this sample was 80%, the tissue necrosis present in this sample may explain this result. Moreover, interpretation of IHC may be complicated for some cases. In our data, 30% of IHC scores were 0+/1+ scoring because of the difficulty to distinguish control nonspecific (0+) staining from cytoplasmic staining of tumoral cells (+1). Moreover, in our study, scoring has been made by only one pathologist whereas some studies showed that a highly reproducible and accurate IHC scoring can be achieved in challenging specimens using stringent IHC criteria and consensus review [20].

Finally, NGS bioinformatics may also been a parameter that may influence the final result. In our study, we decided to analyze the data using 3 different bioinformatics pipelines as described in the material and methods section. No difference has been found using the 3 different pipelines.

The complex mutation c.1796_1799delinsTAGA can be considered as two mutations c.1796C>T (p.Thr599Ile) and c.1799T>A (p.Val600Glu) and was found with all the assays because of the presence of the p.Val600Glu mutation. Response to anti-BRAF therapies in patients with metastatic melanoma bearing complex mutations or single mutation other than V600 have already been described in the literature [21–24] but only p.V600 mutations are officially recognized as response biomarkers for the use of vemurafenib and dabrafenib. NGS and HRM assays allow the extensive detection of all *BRAF* exon 15 including off-hot-spot mutations whose anti-BRAF response prediction value has not been validated. On the other hand, specific BRAF V600 assays (RT-ASA, Idylla^TM^ and IHC) are more restrictive but are designed to match with drugs registration.

Finally, time from sample qualification to *BRAF* mutation analysis result including running-time was 95, 290, 320, 940 and 2,200 minutes for Idylla^TM^, HRM, RT-ASA, IHC and NGS respectively (data not shown). Total hands-on time was 2, 210, 210, 10 and 1,780 minutes for Idylla^TM^, HRM, RT-ASA, IHC and NGS respectively. Idylla^TM^ assay is the fastest assay with a total time of about 95 minutes per sample, but one sample must be assessed in one cartridge inserted in one instrument at time. Up to 8 instruments can be connected to one console allowing the simultaneous detection of 8 samples. In our study, we used two instruments connected to one console, thus this configuration allows the assessment of BRAF mutations in a routine configuration but is not the most appropriate assay for a large batch assessment.

In conclusion, in the present study HRM appears the less sensitive assay for the detection of *BRAF* V600 mutations. The RT-ASA and Idylla^TM^ assays are fully suitable for routine PCR-based molecular diagnostics aiming at the prescription of anti-BRAF therapies. The NGS allows the extensive detection and characterization of all exon 15 *BRAF* mutations, but lacks usefulness until now since no off-hotspots mutations have been described as predictive markers for anti-BRAF therapies. Finally, the Idylla^TM^ assay is easier and faster because it requires less than 2 minutes for sample preparation and all the other steps are fully-automated and assessed in about 90 minutes.
